# A Novel Chiller Sensors Fault Diagnosis Method Based on Virtual Sensors

**DOI:** 10.3390/s19133013

**Published:** 2019-07-08

**Authors:** Long Gao, Donghui Li, Ding Li, Lele Yao, Limei Liang, Yanan Gao

**Affiliations:** 1 School of Electrical and Information Engineering, Tianjin University, Nankai District, Tianjin 300072, China; 2 School of Control Science and Engineering, Shandong University, Lixia District, Jinan 250061, Shandong, China

**Keywords:** virtual sensors, fault detection and diagnosis (FDD), maximum information coefficient (MIC), low false alarm rate, air-cooled chiller

## Abstract

Sensor fault detection and diagnosis (FDD) has great significance for ensuring the energy saving and normal operation of the air conditioning system. Chiller systems serving as an important part of central air conditioning systems are the major energy consumer in commercial and industrial buildings. In order to ensure the normal operation of the chiller system, virtual sensors have been proposed to detect and diagnose sensor faults. However, the performance of virtual sensors could be easily impacted by abnormal data. To solve this problem, virtual sensors combined with the maximal information coefficient (MIC) and a long short-term memory (LSTM) network is proposed for chiller sensor fault diagnosis. Firstly, MIC, which has the ability to quantify the degree of relevance in a data set, is applied to examine all potentially interesting relationships between sensors. Subsequently, sensors with high correlation are divided into several groups by the grouping thresholds. Two virtual sensors, which are constructed in each group by LSTM with different input sensors and corresponding to the same physical sensor, could have the ability to predict the value of physical sensors. High correlation sensors in each group improve the fitting effect of virtual sensors. Finally, sensor faults can be diagnosed by the absolute deviation which is generated by comparing the virtual sensors’ output with the actual value measured from the air-cooled chiller. The performance of the proposed method is evaluated by using a real data set. Experimental results indicate that virtual sensors can be well constructed and the proposed method achieves a significant performance along with a low false alarm rate.

## 1. Introduction

Poorly maintained and improperly controlled equipment wastes an estimated 15% to 30% of energy used in commercial buildings [1]. Heating, ventilation, and air conditioning (HVAC), which maintain comfortable and healthy indoor thermal environments, is an important part of public and private buildings [2]. As an important part of central air conditioning systems, the chiller is the major energy consumer [3]. Hence, it is of vital importance to have a fault detection and diagnosis (FDD) method to maintain optimal operation for chiller systems.

FDD provides a cornerstone for the condition-based maintenance of engineered systems and has been an active area of research and development in the aerospace, process controls, automotive, manufacturing, nuclear, and national defense fields [4,5,6,7,8]. There are many methods to realize FDD in different fields. Data driven methods, rule based methods and model based methods have been used in building systems [9,10,11].

With the rapid development of the computer’s calculation ability, data driven methods have been widely developed in recent years. Han et al. present a novel FDD strategy, which combines the principle component analysis (PCA) feature extraction technology and the multiclass support vector machine (SVM) classification algorithm for vapor-compression refrigeration systems [12]. In order to fully capture the data characteristics, Li proposed a novel data-temporal attention network based strategy for the fault diagnosis of chiller sensors [13]. Fan et al. present a back-propagation neural network black box model using wavelet analysis and fuzzy logic to detect and diagnose faults in an air handling unit [14]. In Reference [15], a dynamic model combined with a data driven method is used to estimate the remaining useful life which does not require prior knowledge of the degradation phenomena.

In another respect, hardware redundancy was first developed to diagnose faults. It has high reliability and can directly isolate faults [16]. Regrettably, hardware redundancy leads to costly and time-consuming processes [17]. To solve this problem, virtual sensors have been proposed for FDD [18] and applied to many fields [19,20,21,22]. Virtual sensors, which are able to estimate various phenomena that are difficult or expensive to measure, can be mass-produced by using black box or gray box models along with other existing physical sensors in building systems [23]. Li et al., review virtual sensing techniques and early applications for buildings [24]. In building systems, virtual sensors can be constructed with historical data which are stored by a monitoring system. The method proposed in Reference [18] is applied to detect and diagnose faults in ventilation units using virtual sensors. The physical sensors can be predicted by virtual sensors with satisfactory accuracy. The method proposed in Reference [25] is applied to exploit physical relations inside the unit using linear regression virtual sensors. Vasso et al. present a local virtual sensor agent for diagnosing sensor faults in HVAC systems, and compensating for their effects on the distributed control architecture [26]. Kusiak et al. have constructed sensor models for predicting temperature, CO${}_{2}$, and relative humidity by data mining algorithms. It can be applied to HVAC systems in various buildings [27]. However, all of the above virtual sensors’ performances could be easily impacted by input parameters, such as historical data of a building system and external factors [23].

In this paper, a novel chiller sensor fault diagnosis method based on a virtual sensor technique and a data driven method is proposed. Firstly, a large amount of sensor data can be stored when the chiller system is operating. According to the data collected from the chiller system, sensors can be divided into several groups by the maximal information coefficient (MIC) and the sensor grouping threshold. MIC is used to explore relationships among different sensors, which can better describe the nonlinear relationship in data set than traditional methods such as Pearson correlation. Sensors in each group have high correlation which is helpful for improving the performance of the virtual sensors. Subsequently, virtual sensors are constructed by a long short-term memory network (LSTM) in each group. Deep learning models are capable of automatic and deep mining feature information, which have made achievements in many fields [28,29,30]. As a deep learning model, LSTM could be capable of automatic and deep mining feature information in sensor data, which can better improve the performance of virtual sensors. In the end, the trained networks could properly fit the chiller system. The fault can be diagnosed by the absolute deviation which is generated by comparing the predicted output of the virtual sensors with the actual value.

The contributions of the paper are as follows:

1. In order to ensure the performance of virtual sensors, MIC is used to examine potentially interesting relationships between sensors. Chiller sensors with high MIC scores are divided into the same groups. This could dramatically improve the fitting effect of virtual sensors by constructing them in the same group.
2. The performance of virtual sensors could be easily impacted by the input sensors. In order to reduce the false alarm rate, two virtual sensors that have different input sensors are constructed for the same physical sensor. When the two deviations between the corresponding physical sensor and the two virtual sensors both exceed the thresholds, the physical sensor is considered as a fault state.
3. The LSTM model, which can better extract discriminating features from the sensor data, is used to construct the virtual sensors. It could further improve the fitting effect of virtual sensors.

The rest of this paper is organized as follows: Section 2 gives the description and coupling characteristic analysis of the air-cooled chiller system; the proposed chiller sensor fault diagnosis method is introduced in Section 3; Section 4 verifies the effectiveness of the proposed method on the sensor fault diagnosis performance along with low false alarm rate; and Section 5 draws the conclusions.

## 2. Coupling Characteristic Analysis of the Air-Cooled Chiller System

**Figure 1:**
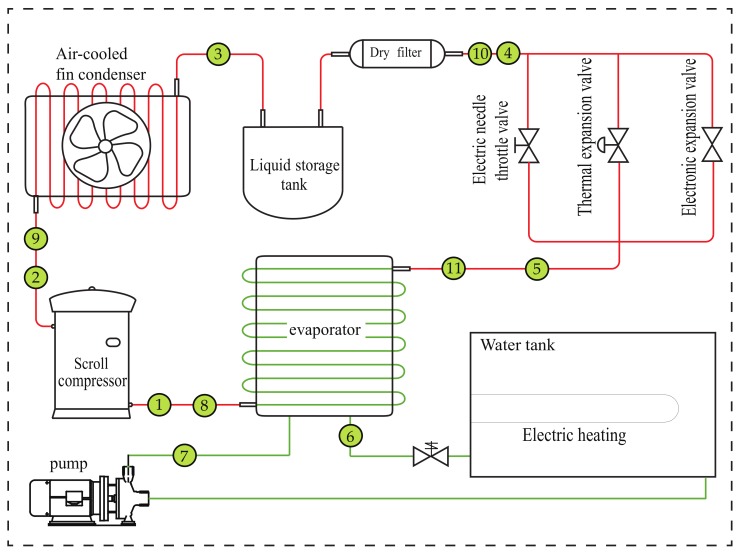
Schematic diagram of the air-cooled chiller system. The system consists of refrigerant closed loop and chilled water closed loop.

Figure 1 shows the schematic of an existing air-cooled chiller system. The main equipment of the air-cooled chiller includes a scroll compressor, air-cooled condenser, throttle device and evaporator. The throttling device is provided with three different throttling modes: electronic expansion valve, thermal expansion valve and electric needle throttle. This paper mainly uses an electronic expansion valve to throttle the refrigerant. The chilled water, which is powered by the pump, circulates by the green line. When the water temperature in the water tank is lowered, the electric heater, which maintains the temperature of the water tank, is used to compensate the heat consumption.

The heat is transferred from the low-temperature heat source to the high-temperature heat source via refrigerant circulation loop. In the circulation loop of the refrigerant, the energy conservation can be written as

(1) $$Q_{e}+Q_{com}=Q_{c},$$

where $Q_{com}$ represents the electric power consumed by the compressor, $Q_{e}$ represents the amount of cold generated in the evaporator and $Q_{c}$ represents the heat released by the condenser.

The refrigerant circulates via the compressor, condenser, expansion valve and evaporator while performing energy transfer with the chilled water. The pressure, enthalpy and other parameters of the refrigerant in the condenser can be affected by the output parameters of the compressor, the outlet refrigerant enthalpy, the mass flow rate, and so forth. Conversely, the compressor power and the refrigerant mass flow can be changed by the compressor input parameters such as the condenser condensing pressure. The parameters, such as the electronic expansion valve mass flow and the outlet enthalpy, can be affected by the output parameters of the condenser, the condensing pressure, the outlet refrigerant enthalpy, and so forth. The condensing pressure can also be affected by the electronic expansion valve mass flow. The electronic expansion valve output parameters and the enthalpy affect the evaporation pressure, the outlet enthalpy and other parameters. The evaporation pressure as the expansion valve input can also affect the electronic expansion valve mass flow. There is also a coupling relationship between the compressor and the evaporator through the evaporation pressure and the mass flow of the compressor. Thus, there is a complicated coupling relationship between the various equipment of the chiller system.

## 3. Methods

### 3.1. Maximal Information Coefficient

Due to the complex coupling characteristic of the air-cooled chiller system, MIC which has the ability to examine all potentially interesting relationships is used to explore relationships among different sensors. The calculation of MIC is based on mutual information (MI). MI tells us how much knowing one variable reduces our uncertainty about the other [31]. And the mutual information of two variables *X* and *Y* can be defined as

(2) $$I\left(X,Y\right)=\int \int u\left(x,y\right)log\frac{u(x,y)}{u_{x}\left(x\right)u_{y}\left(y\right)}\;dxdy,$$

where u(x,y) is the joint probability density function of *X* and *Y*, $u_{x}\left(x\right)=\int u\left(x,y\right)\;dy$ and $u_{y}\left(y\right)\;=\;\int u\left(x,y\right)\;dx$.

In order to get the MIC score, MI is first achieved by exploring all the grids by using different partition schemes, then the MIC is normalized between 0 and 1 by dividing $log_{2}\left(min\left(X,Y\right)\right)$, and the maximum is chosen as the MIC score. Thus the MIC score can be calculated as [32]

(3) $$M\left(D\right)=\max_{XY<B(n)}\frac{I(D,X,Y)}{log_{2}\left(min\left(X,Y\right)\right)},\;$$

where *B* is a function of sample size *n*, usually $B=n^{0.6}$; I(D,X,Y) is the maximum mutual information value that falls into the mesh region *D*. Variables *X* and *Y* are independent of each other when MIC is equal to 0. And some kinds of functional relationship are existed between *X* and *Y* variables when MIC is equal to 1.

Raw operating data, which include eleven sensors, compressor frequency $C_{s}$ and electron expansion valve $E_{ev}$, are provided by the air-cooled chiller system. In this system, temperature is measured using PT-100, whose average error is around 0.3 ${}^{\circ }$C. The pressure on the scroll compressor and throttle device are measured by pressure transducers with the accuracy 0.5%. The descriptions of the eleven sensors have been listed in Table 1.

**Figure 2:**
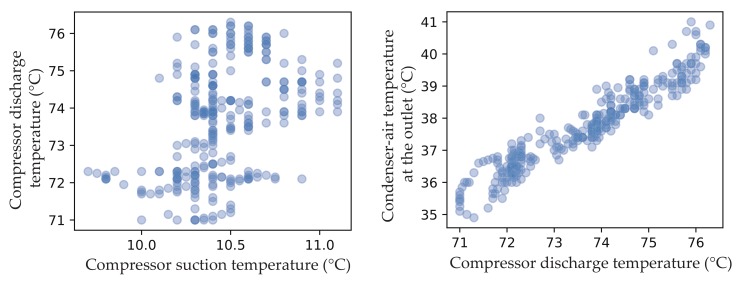
Density plots of $T_{suc}$ and $T_{dis}$, $T_{dis}$ and $T_{con\_out}$.

The maximum mutual information is calculated between any sensors in Table 2. Multiple chiller sensors are divided in the same group to ensure the correlation between the sensors, which the MIC score is greater than grouping threshold. For instance, sensors can be divided into {$T_{suc}$, $T_{chw\_s}$, $T_{chw\_r}$, $P_{th\_out}$} and {$P_{suc}$, $T_{con\_out}$, $T_{ref\_b}$, $P_{dis}$} for virtual senors $T_{suc}$ and $P_{suc}$, when the grouping threshold takes 0.8. In order to verify MIC mining relationship capabilities between the sensors, density plots of $T_{suc}$ and $T_{dis}$, $T_{dis}$ and $T_{con\_out}$ are shown in Figure 2. As we can see, $T_{suc}$ and $T_{dis}$ which the MIC score is equal to 0.239 are independent on each other, while $T_{dis}$ and $T_{con\_out}$ which the MIC score is equal to 0.812 are obviously highly correlated.

### 3.2. Virtual Sensors

**Figure 3:**
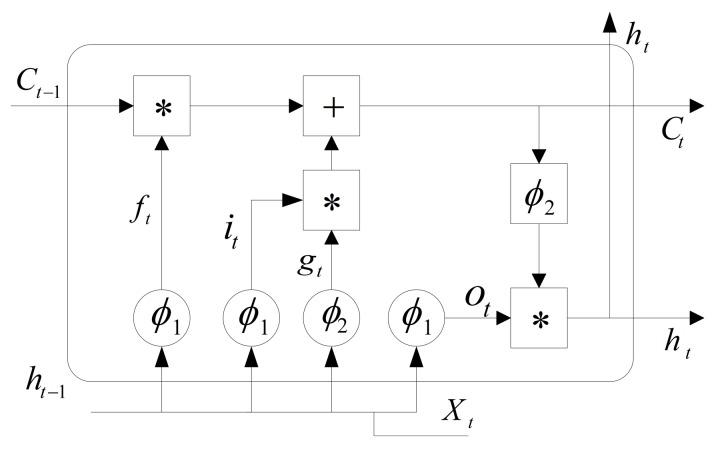
LSTM cell.

The LSTM model is used to construct the virtual sensors for eleven physical sensors. It can better extract feature information among different sensors in the chiller system. As shown in Figure 3, the key to LSTM is the LSTM cell which can decide whether to maintain state information from the prior step. LSTM cells contain input gate, forget gate and output gate. The input gate is used to decide the input information to save the state of unit. The forget gate is used to decide the state of unit at the last time. The output gate is used to decide the output of LSTM cell. Outputs $c_{t}$ and $h_{t}$ are recurrently connected to the inputs of block. The LSTM cell with forward propagation is calculated as follows:

(4) $$f_{t}=\sigma \left(W_{f}*\left[h_{t-1},x_{t}\right]+b_{f}\right),$$

(5) $$i_{t}=\sigma \left(W_{i}*\left[h_{t-1},x_{t}\right]+b_{i}\right),$$

(6) $$o_{t}=\sigma \left(W_{o}*\left[h_{t-1},x_{t}\right]+b_{o}\right),$$

(7) $$c_{t}=f_{t}*c_{t-1}+i_{t}*g_{t},$$

(8) $$g_{t}=tanh\left(W_{c}*\left[h_{t-1},x_{t}\right]+b_{c}\right),$$

(9) $$h_{t}=o_{t}*tanh\left(c_{t}\right).$$

The Formulas (4)–(6) are forget gate, input gate and output gate respectively. $W_{f}$, $W_{i}$ and $W_{c}$ are the weights of forget gate, input gate and output gate respectively. σ(·) and tanh(·) are the sigmoid function and hyperbolic tangent respectively.

**Figure 4:**
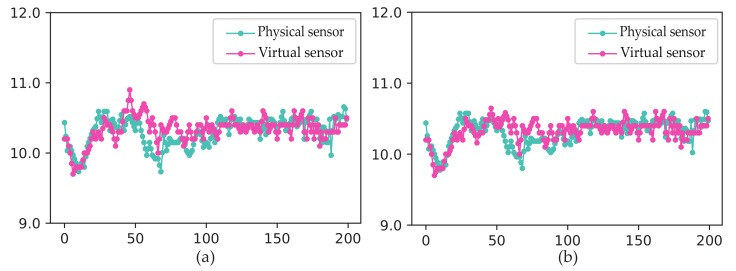
(**a**) Predictive result of virtual sensor $V_{T_{suc}}^{1}$. (**b**) Predictive result of virtual sensor $V_{T_{suc}}^{2}$.

Herein, the input of the LSTM model includes the sensor historical data in same group, compressor frequency and electron expansion valve. And the output of the LSTM model constructs corresponding virtual sensors. Two virtual sensors, which contain different input sensors and correspond to the same physical sensor, are constructed in the same group. The maximum correlation ensures the performance of the two virtual sensors with different input physical sensors. For instance, if the grouping threshold takes 0.3 in Table 3, the chiller sensors can be divided into two groups: {$T_{suc}$, $T_{ref\_a}$, $T_{chw\_s}$, $T_{chw\_r}$, $P_{th\_in}$, $P_{th\_out}$} and {$P_{suc}$, $T_{dis}$, $T_{con\_out}$, $T_{ref\_b}$, $T_{chw\_s}$, $T_{chw\_r}$, $P_{dis}$}. Virtual sensor $T_{suc}^{\prime }$ and $T_{suc}^{\prime \prime }$ for $T_{suc}$ are constructed as

(10) $$V_{T_{suc}}^{\prime }=f\left(\vec{V_{T_{suc}}^{1}}\right),$$

(11) $$V_{T_{suc}}^{\prime \prime }=f\left(\vec{V_{T_{suc}}^{2}}\right),$$

(12) $$\vec{V_{T_{suc}}^{1}}=\left(T_{ref\_a},T_{chw\_s},T_{chw\_r},C_{s},E_{ev}\right),$$

(13) $$\vec{V_{T_{suc}}^{2}}=\left(P_{th\_in},P_{th\_out},C_{s},E_{ev}\right),$$

where $\vec{V_{T_{suc}}^{1}}$ and $\vec{V_{T_{suc}}^{2}}$ are acquired sequentially from the corresponding groups and represents the sensors historical data. $V_{T_{suc}}^{\prime }$ and $V_{T_{suc}}^{\prime \prime }$ represent corresponding virtual sensors and f(·) represents LSTM model. The actual value and predictive value for $T_{suc}$ are shown in Figure 4.

### 3.3. The Threshold and Procedure of Fault Diagnosis

In the actual implementation, the fault can be diagnosed by the absolute deviation between the virtual sensors and the physical sensors. A fault diagnosis threshold is calculated to automatically diagnose sensor fault. The fault diagnosis threshold of the *l*-th sensor is calculated as

(14) $$V_{FD}^{l}=\max_{p\in S_{T}}\left(abs\left(v_{p}^{l}-a_{p}^{l}\right)\right),$$

where $v_{p}^{l}$ is the predictive value of virtual sensors, $a_{p}^{l}$ is the actual value of chiller sensors and $S_{T}$ is a set to determinate fault diagnosis threshold. Different group thresholds can be obtained for different sensors.

**Figure 5:**
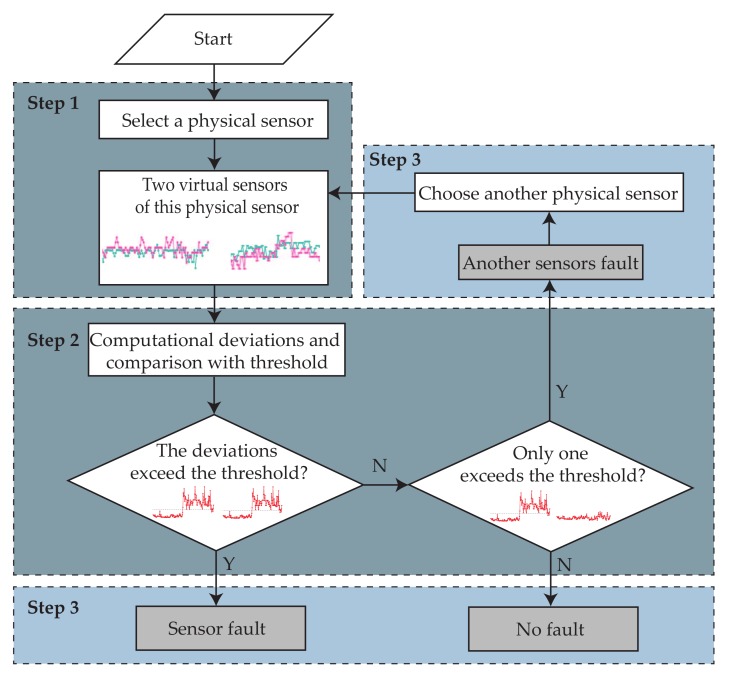
Flow chart of fault diagnosis.

Figure 5 shows the flow chart of fault diagnosis. The proposed fault diagnosis method for chiller system includes three steps:

**Step 1:** 
A physical sensor is selected from eleven sensors. Two virtual sensors, which have been constructed in the training period, are used to predict the value of this physical sensor.
**Step 2:** 
Deviations between virtual and physical sensors are calculated and compared with the fault diagnosis threshold.
**Step 3:** 
Obviously, the physical sensor is not considered as a fault state when no deviations exceed the threshold. On the contrary, a sensor fault occurs when the deviations both exceed the fault diagnosis threshold. Input sensors are considered as a fault state if only one absolute deviation exceeds the threshold, because two virtual sensors have different input sensors. Under this situation, another physical sensor from input sensors is selected and step 2 will be repeated to predict the value of another physical sensor.

## 4. Results and Discussion

### 4.1. Experimental Data

The experimental data are acquired from the device in the Honeywell Home and Building Control Laboratory in Tianjin University, China. During the normal operation of the device, the chiller system can store thirteen kinds of measurement data such as different sensor values and electronic expansion valve opening degree which are highly correlated with the sensor reading. The experimental data, which obtain 45,000 sets of time series, are collected at 2 min intervals and divided into training sets, fault diagnosis threshold determination sets and test sets.

Before the experiment began, the raw data need to be standardized and filter out samples with poor quality, such as those during chiller start up and shutdown time periods. Based on the measurement accuracy of temperatures and pressure sensors, eleven sensor faults are introduced by adding the fixed bias in this paper. Various magnitudes of biases, which are determined by the reading range of each sensor, have been added to each sensor in Table 4.

### 4.2. Performance Comparison

To verify the low false alarm rate and fault diagnosis performance of this method, $T_{suc}$ and $P_{suc}$ are chosen as instances. As described in Section 3.1, different groups for $T_{suc}$ and $P_{suc}$ have been obtained and listed in Table 3 when different grouping thresholds are used. As can be seen, the number of the sensors has decreased along with the increase of the grouping thresholds. A high correlation between sensors will contribute to the constructing of a virtual sensor in the same group. All chiller sensors are divided into the same group, if the grouping threshold takes 0.

#### 4.2.1. Verification of Low False Alarm Rate

**Figure 6:**
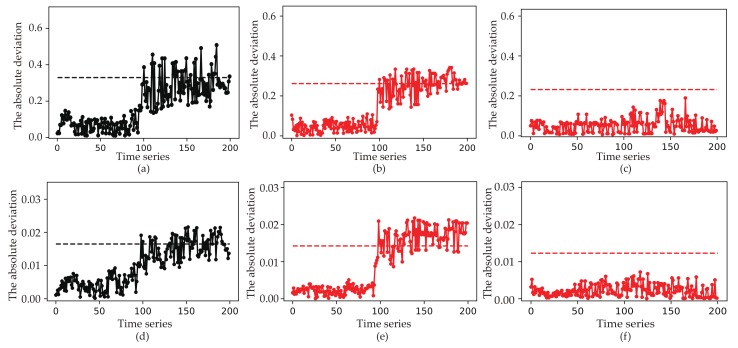
The absolute deviation of $T_{suc}$ and $P_{suc}$ with different virtual sensors. (**a**) The absolute deviation between the physical sensor $T_{suc}$ and the only virtual sensor. (**b**) The absolute deviation between $T_{suc}$ and the first of the two virtual sensor. (**c**) The absolute deviation between $T_{suc}$ and the second of the two virtual sensor after grouping. (**d**) The absolute deviation between the physical sensor $P_{suc}$ and the only virtual sensor. (**e**) The absolute deviation between $P_{suc}$ and the first of the two virtual sensor. (**f**) The absolute deviation between $P_{suc}$ and the second of the two virtual sensor.

We assume that only one sensor is considered as a fault state. Input sensors $T_{chw\_s}$ and $T_{con\_out}$ with minimum biases are used to verify the low false alarm rate of this method. Sensor grouping threshold takes 0.8. In the figure below, the first half time series are normal samples and the last half are fault samples. Figure 6 shows the absolute deviation of $T_{suc}$ and $P_{suc}$ when the corresponding input sensors $T_{chw\_s}$ and $T_{con\_out}$ are faulty. As can be seen in Figure 6a, if only one virtual sensor is used, the absolute deviation will increases after fault occurs. It means that the fault diagnosis performance will be affected by the input sensor. However, another sensor fault occurs if only one absolute deviation exceeds the fault diagnosis threshold, as depicted in Figure 5 and Figure 6b,c. The false alarm rate of $T_{suc}$ is reduced from 28.0% to 0.0%. In the same way, the false alarm rate of $P_{suc}$ is reduced from 41.0% to 0.0%.

To further verify the performance of this method, the false alarm rate of all chiller sensors is listed in Table 5. As we can see, the false alarm rate is close to 0 when two virtual sensors with different input sensor are used. For instance, the false alarm rate of $T_{con\_out}$ will reduce from 18.0% to 3.0%, if two virtual sensors for $T_{con\_out}$ are used. The high false alarm rate will occur if only one virtual sensor is used. Therefore, two virtual sensors with different input sensors can achieve a low false alarm rate.

#### 4.2.2. Fault Diagnosis Performance

As a deep learning model, the LSTM model, which is developed to address sequential data with its ability to encode temporal information, has a better performance than the linear regression and nonlinear regression models such as the artificial neural network model. Linear regression is a linear approach to modeling the relationship among different sensors. The artificial neural network model, which has great nonlinear curve fitting capability, can achieve a better performance than the linear regression model. Due to the high correlation between sensors in the same group and the strong feature extract ability of the LSTM model, either of the two sensors can achieve a better performance on fault diagnosis. Virtual sensors are used to diagnose faults in this paper, which are constructed by the linear regression (LR) model, the artificial neural network (ANN) model and the LSTM model. $T_{suc}$ and $P_{suc}$ with minimum biases are chosen as instances.

**Figure 7:**
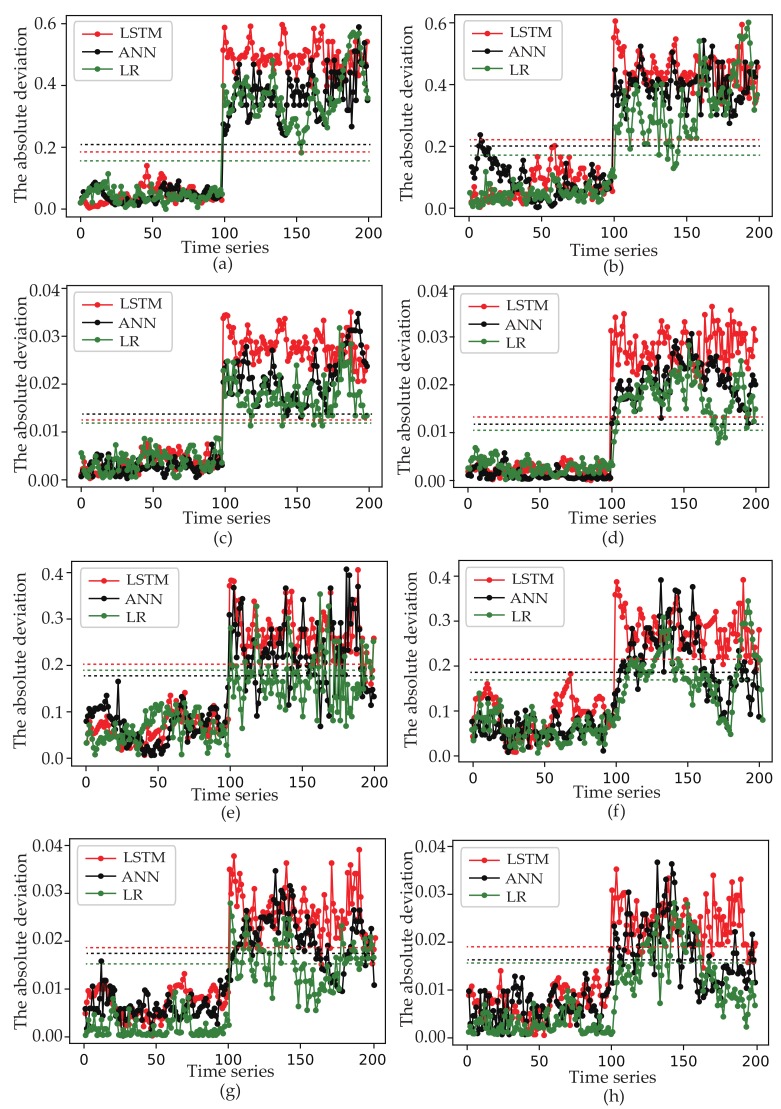
The absolute deviation of $T_{suc}$ and $P_{suc}$. (**a**) The absolute deviation of virtual sensor $T_{suc}^{\prime }$ when the grouping threshold takes 0.8; (**b**) The absolute deviation of virtual sensor $T_{suc}^{\prime \prime }$ when the grouping threshold takes 0.8; (**c**) The absolute deviation of virtual sensor $P_{suc}^{\prime }$ when the grouping threshold takes 0.8; (**d**) The absolute deviation of virtual sensor $P_{suc}^{\prime \prime }$ when the grouping threshold takes 0.8; (**e**) The absolute deviation of virtual sensor $T_{suc}^{\prime }$ when the grouping threshold takes 0; (**f**) The absolute deviation of virtual sensor $T_{suc}^{\prime \prime }$ when the grouping threshold takes 0; (**g**) The absolute deviation of virtual sensor $P_{suc}^{\prime }$ when the grouping threshold takes 0; (**h**) The absolute deviation of virtual sensor $P_{suc}^{\prime \prime }$ when the grouping threshold takes 0.

The performance of virtual sensors can be verified by the different grouping thresholds. In Figure 7, the absolute deviation and fault diagnosis ratios obviously increase along with increase of grouping threshold. The absolute deviation of the LSTM-based virtual sensor has significant performance than FC-based and LR-based virtual sensor, when the fault samples occur. For instance, the average absolute deviation between pressure sensor $P_{suc}$ and virtual sensors will decrease from 0.027 to 0.021, if LSTM-based virtual sensor is replaced by FC-based and the grouping threshold takes 0.8. The average absolute deviation is decreased from 0.027 to 0.019 and the fault diagnosis ratios is decreased from 100.0% to 95.0% if the LSTM-based virtual sensor is similarly replaced by LR-based. When the fault samples occur, increasing absolution deviation can be obtained along with the increase of the grouping thresholds. For instance, the average absolute deviation is decreased from 0.021 to 0.027, if the grouping threshold takes 0 instead of 0.8. It reveals that LSTM-based virtual sensors have an excellent performance along with the increase of the grouping threshold.

**Figure 8:**
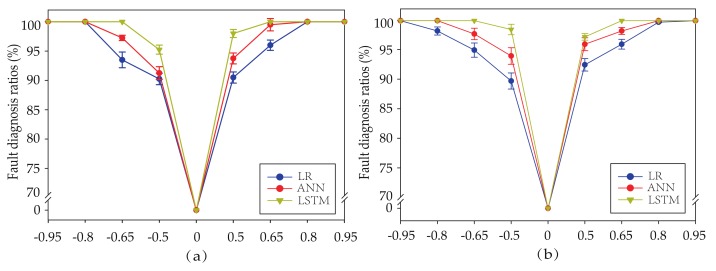
(**a**) Average fault diagnosis ratio of temperature sensor $T_{suc}$ with different biases. (**b**) Average fault diagnosis ratio of temperature sensor $P_{suc}$ with different biases.

As shown in Figure 8, average fault diagnosis ratio are compared in different biases. For these two sensors, the fault diagnosis ratios of three methods increases with the increase of the introduced biases fault in Table 4. All methods easily reach 100% when the magnitude of biases is the maximum of each sensor. However, as the magnitude of biases decreases, the fault diagnosis rates show a difference. For instance, when the biases of the temperature sensor $T_{suc}$ is tiny, such as 0.5, FC-based and LR-based virtual sensors have a poor performance while the fault diagnosis ratio of the LSTM-based virtual sensor reaches 98%. It means that LSTM-based virtual sensors have a better performance in the case of the positive or negative biases. When the biases are negative, all methods are influenced to various extents. For instance, average fault diagnosis ratios of the pressure sensor $P_{suc}$ are 98.5%, 94% and 89.75% respectively, provided that magnitude of biases is changed from 0.5 to −0.5. However, fault diagnosis ratios of LSTM-based virtual sensors always achieve a better performance than other methods.

To further verify the performance of another virtual sensor during training and testing, the mean absolute error (MAE) is used in this paper. MAE is frequently used to measure the differences between values predicted by a model and the values observed. The MAE between the physical sensor and virtual sensor is defined as

(15) $$MAE=\frac{1}{N}\sum_{i=1}^{N}\left(x_{i}-\hat{x_{i}}\right),$$

where *N* represents sampling steps, $x_{i}$ represents value of virtual sensor and $\hat{x_{i}}$ represents value of physical sensor. In actual implementation, the MAE of the corresponding sensor is obtained by calculating the average of the two virtual sensors.

As shown in Table 6, *p*-values between the traditional methods and the proposed method were calculated. The *t* test can easily be adapted to testing the following hypotheses at a specified level of significance $\alpha_{0}$: $H_{0}:\mu_{1}=\mu_{2}$, $H_{1}:\mu_{1}\neq \mu_{2}$. $H_{0}$ will be rejected at any level $\alpha_{0}\geq 0.05$, which means there is not a significant difference between the two methods. As we can see, *p*-values between the traditional methods and the proposed method are always smaller than 0.05. This means that LSTM-based virtual sensors are significantly different to the two other methods.

**Figure 9:**
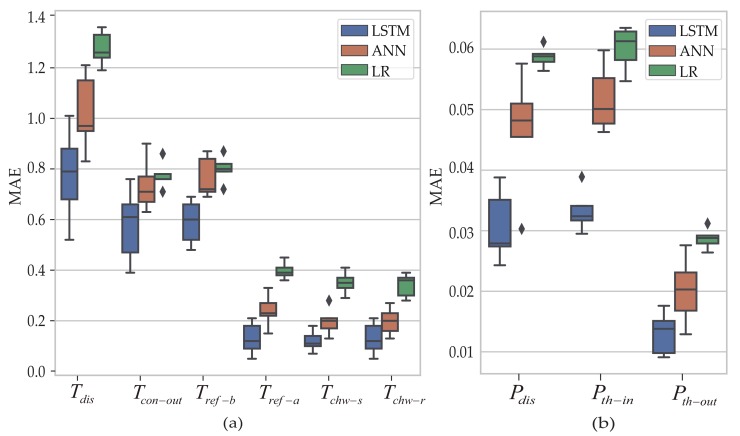
(**a**) MAE of the remaining temperature sensors during training. (**b**) MAE of the remaining pressure sensors during training.

In addition to $T_{suc}$ and $P_{suc}$, Figure 9 shows MAE of another nine sensors during training when the grouping threshold takes 0.8. Smaller value of MAE indicates better training performance of the virtual sensors. As we can see, due to the different reading range of different sensors, virtual sensors show different performances for different physical sensors. However, LSTM-based virtual sensors always have a lower MAE value compared with LR-based or FC-based virtual sensors. It reveals that LSTM-based virtual sensors always have a better training performance. Table 7 summarizes MAE of another nine sensors during testing, to which minimum positive biases are added. The grouping threshold also takes 0.8. On the contrary, the maximal value of MAE indicates better training performance of the virtual sensors. When the minimum negative bias occurred, virtual sensors based on different models show a different performance. Therefore, LSTM-based virtual sensors have a better performance for both positive and negative biases.

In order to verify the performance of different physical sensors, fault diagnosis ratios of all physical sensors with minimum biases are listed in Table 8 and Table 9. Table 8 lists fault diagnosis ratios when the grouping threshold takes 0.8 and the minimum positive biases are added. It can be clearly seen that LSTM-based virtual sensors have a better performance than another two methods. This conclusion is consistent with the results of Figure 9 and Table 7. For instance, fault diagnosis rate with positive bias is 91.00% and 96.25% rather than 98.75%, when the LSTM-based virtual sensor is similarly replaced by LR-based and FC-based virtual sensor for physical sensor $T_{con\_out}$. The fault diagnosis ratios are reduced by 7.75% and 2.5% compared to LR-based and FC-based virtual sensor, respectively. When the minimum negative biases occur, LSTM-based virtual sensors still have a better performance in Table 9.

## 5. Conclusions

The operating efficiency of an air-cooled chiller system is critical for building energy performance. In this paper, a novel chiller sensor fault diagnosis method was proposed. Chiller sensors were divided into different groups by MIC score and the grouping threshold. Highly correlated sensors can significantly improve the accuracy of virtual sensors. Under the same model, experimental results also show that virtual sensors have a better performance by grouping with the MIC score. Virtual sensors constructed by LSTM model were compared with the linear regression and the artificial neural network model, which reduces the cost of hardware redundancy. In order to reduce the false alarm rate and directly diagnose sensor faults, two virtual sensors corresponding to the same physical sensor were constructed. Comparing the method in this paper with using only one virtual sensor, the method in this paper has the lower false alarm rate. The proposed method with higher accuracy has also been well presented and experimentally validated.

The following work needs to be done in the future: firstly, fault diagnosis threshold determination is an indispensable part of the chiller sensor fault diagnosis based on deviation. Threshold will directly impact the performance of fault diagnosis. An excellent method for fault diagnosis threshold should be further researched; secondly, the proposed method will be applied to other refrigeration air conditioning systems to further verify generalization.

## Figures and Tables

**Table 1:** The descriptions of the eleven sensors.

No.	Sensors	Descriptions	Unit
1	$T_{suc}$	Compressor suction temperature	${}^{\circ }$C
2	$T_{dis}$	Compressor discharge temperature	${}^{\circ }$C
3	$T_{con\_out}$	Condenser-air temperature at the outlet	${}^{\circ }$C
4	$T_{ref\_b}$	Refrigerant temperature before throttling	${}^{\circ }$C
5	$T_{ref\_a}$	Refrigerant temperature after throttling	${}^{\circ }$C
6	$T_{chw\_s}$	Chilled-water supply temperature	${}^{\circ }$C
7	$T_{chw\_r}$	Chilled-water return temperature	${}^{\circ }$C
8	$P_{suc}$	Compressor suction pressure	MPa
9	$P_{dis}$	Compressor discharge pressure	MPa
10	$P_{th\_in}$	Inlet pressure of the throttle device	MPa
11	$P_{th\_out}$	Outlet pressure of the throttle device	MPa

**Table 2:** MIC of different sensors.

	$T_{\mathrm{suc}}$	$T_{\mathrm{dis}}$	$T_{\mathrm{con}\_\mathrm{out}}$	$T_{\mathrm{ref}\_b}$	$T_{\mathrm{ref}\_a}$	$T_{\mathrm{chw}\_s}$	$T_{\mathrm{chw}\_r}$	$P_{\mathrm{suc}}$	$P_{\mathrm{dis}}$	$P_{\mathrm{th}\_\mathrm{in}}$	$P_{\mathrm{th}\_\mathrm{out}}$
$T_{suc}$	1	0.239	0.264	0.279	0.432	0.850	0.881	0.267	0.271	0.618	0.832
$T_{dis}$	0.239	1	0.812	0.652	0.307	0.237	0.237	0.750	0.827	0.319	0.092
$T_{con\_out}$	0.264	0.812	1	0.836	0.249	0.305	0.299	0.922	0.898	0.255	0.160
$T_{ref\_b}$	0.279	0.652	0.836	1	0.289	0.337	0.328	0.864	0.906	0.273	0.188
$T_{ref\_a}$	0.432	0.307	0.249	0.289	1	0.348	0.422	0.255	0.263	0.906	0.805
$T_{chw\_s}$	0.850	0.237	0.305	0.337	0.348	1	0.886	0.334	0.333	0.335	0.516
$T_{chw\_r}$	0.881	0.237	0.299	0.328	0.422	0.886	1	0.325	0.328	0.431	0.499
$P_{suc}$	0.267	0.750	0.922	0.864	0.255	0.334	0.325	1	0.940	0.260	0.180
$P_{dis}$	0.271	0.827	0.898	0.906	0.263	0.333	0.328	0.940	1	0.262	0.183
$P_{th\_in}$	0.618	0.319	0.255	0.273	0.906	0.335	0.431	0.260	0.262	1	0.135
$P_{th\_out}$	0.832	0.092	0.160	0.188	0.805	0.516	0.499	0.180	0.183	0.135	1

**Table 3:** Different groups for $T_{suc}$ and $P_{suc}$.

Grouping Threshold	Group
0.8	{$T_{suc}$, $T_{chw\_s}$, $T_{chw\_r}$, $P_{th\_out}$} {$P_{suc}$, $T_{con\_out}$, $T_{ref\_b}$, $P_{dis}$ }
0.6	{$T_{suc}$, $T_{chw\_s}$, $T_{chw\_r}$, $P_{th\_in}$, $P_{th\_out}$} {$P_{suc}$, $T_{dis}$, $T_{con\_out}$, $T_{ref\_b}$, $P_{dis}$ }
0.3	{$T_{suc}$, $T_{ref\_a}$, $T_{chw\_s}$, $T_{chw\_r}$, $P_{th\_in}$, $P_{th\_out}$} {$P_{suc}$, $T_{dis}$, $T_{con\_out}$, $T_{ref\_b}$, $T_{chw\_s}$, $T_{chw\_r}$, $P_{dis}$ }

**Table 4:** Biases added in different sensors.

Sensors	Unit	Biases
$T_{suc}$	${}^{\circ }$C	±0.5, ±0.65, ±0.8, ±0.95
$T_{dis}$	${}^{\circ }$C	±1.5, ±1.9, ±2.3, ±2.7
$T_{con\_out}$	${}^{\circ }$C	±1.0, ±1.3, ±1.6, ±1.9
$T_{ref\_b}$	${}^{\circ }$C	±1.0, ±1.3, ±1.6, ±1.9
$T_{ref\_a}$	${}^{\circ }$C	±0.4, ±0.5, ±0.6, ±0.7
$T_{chw\_s}$	${}^{\circ }$C	±0.4, ±0.5, ±0.6, ±0.7
$T_{chw\_r}$	${}^{\circ }$C	±0.4, ±0.5, ±0.6, ±0.7
$P_{suc}$	MPa	±0.035, ±0.04, ±0.045, ±0.05
$P_{dis}$	MPa	±0.07, ±0.08, ±0.09, ±0.10
$P_{th\_in}$	MPa	±0.07, ±0.08, ±0.09, ±0.10
$P_{th\_out}$	MPa	±0.035, ±0.04, ±0.045, ±0.05

**Table 5:** False alarm rate of chiller sensors.

		$T_{\mathrm{dis}}$	$T_{\mathrm{con}\_\mathrm{out}}$	$T_{\mathrm{ref}\_b}$	$T_{\mathrm{ref}\_a}$	$T_{\mathrm{chw}\_s}$	$T_{\mathrm{chw}\_r}$	$P_{\mathrm{dis}}$	$P_{\mathrm{th}\_\mathrm{in}}$	$P_{\mathrm{th}\_\mathrm{out}}$
	Only one virtual sensor	35.0%	18.0%	26.0%	52.0%	44.0%	27.0%	39.0%	11.0%	23.0%
	Two virtual sensors	0.00%	3.0%	0.00%	0.00%	0.00%	2.0%	4.0%	0.00%	0.00%

**Table 6:** *P*-values between the traditional methods and the proposed method.

	$T_{\mathrm{dis}}$	$T_{\mathrm{con}\_\mathrm{out}}$	$T_{\mathrm{ref}\_b}$	$T_{\mathrm{ref}\_a}$	$T_{\mathrm{chw}\_s}$	$T_{\mathrm{chw}\_r}$	$P_{\mathrm{dis}}$	$P_{\mathrm{th}\_\mathrm{in}}$	$P_{\mathrm{th}\_\mathrm{out}}$
LR-based, LSTM-based	$1.8\times 10^{-5}$	$3.5\times 10^{-3}$	$1.3\times 10^{-2}$	$5.4\times 10^{-4}$	$1.4\times 10^{-2}$	$3.3\times 10^{-3}$	$2.1\times 10^{-3}$	$6.2\times 10^{-4}$	$1.5\times 10^{-3}$
FC-based, LSTM-based	$2.0\times 10^{-2}$	$1.6\times 10^{-2}$	$2.8\times 10^{-4}$	$3.7\times 10^{-4}$	$2.3\times 10^{-2}$	$2.9\times 10^{-3}$	$3.0\times 10^{-2}$	$5.8\times 10^{-4}$	$2.9\times 10^{-3}$

**Table 7:** MAE of the remaining sensors during testing.

		$T_{\mathrm{dis}}$	$T_{\mathrm{con}\_\mathrm{out}}$	$T_{\mathrm{ref}\_b}$	$T_{\mathrm{ref}\_a}$	$T_{\mathrm{chw}\_s}$	$T_{\mathrm{chw}\_r}$	$P_{\mathrm{suc}}$	$P_{\mathrm{th}\_\mathrm{in}}$	$P_{\mathrm{th}\_\mathrm{out}}$
	LR-based	1.37	0.89	0.87	0.42	0.44	0.38	0.061	0.057	0.032
Positive	FC-based	1.08	0.84	0.85	0.29	0.21	0.27	0.049	0.053	0.021
	LSTM-based	0.85	0.68	0.69	0.17	0.19	0.20	0.034	0.038	0.016
	LR-based	1.41	0.82	0.89	0.33	0.47	0.39	0.067	0.062	0.035
Negative	FC-based	1.12	0.89	0.81	0.33	0.27	0.31	0.052	0.058	0.018
	LSTM-based	0.87	0.56	0.75	0.20	0.21	0.25	0.032	0.040	0.019

**Table 8:** Fault diagnosis ratios with positive bias.

	$T_{\mathrm{suc}}$	$T_{\mathrm{dis}}$	$T_{\mathrm{con}\_\mathrm{out}}$	$T_{\mathrm{ref}\_b}$	$T_{\mathrm{ref}\_a}$	$T_{\mathrm{chw}\_s}$	$T_{\mathrm{chw}\_r}$	$P_{\mathrm{suc}}$	$P_{\mathrm{dis}}$	$P_{\mathrm{th}\_\mathrm{in}}$	$P_{\mathrm{th}\_\mathrm{out}}$
LR-based	90.5%	92.25%	91.0%	94.5%	93.25%	89.5%	90.5%	92.5%	93.0%	95.25%	94.5%
FC-based	93.75%	93.5%	96.25%	95.75%	95.5%	91.75%	97.5%	96.0%	95.5%	96.25%	97.75%
LSTM-based	98%	98.5%	98.75%	97.25%	97.75%	96.25%	100.0%	97.25%	99.25%	97.5%	99.5%

**Table 9:** Fault diagnosis ratios with negative bias.

		$T_{\mathrm{suc}}$	$T_{\mathrm{dis}}$	$T_{\mathrm{con}\_\mathrm{out}}$	$T_{\mathrm{ref}\_b}$	$T_{\mathrm{ref}\_a}$	$T_{\mathrm{chw}\_s}$	$T_{\mathrm{chw}\_r}$	$P_{\mathrm{suc}}$	$P_{\mathrm{dis}}$	$P_{\mathrm{th}\_\mathrm{in}}$	$P_{\mathrm{th}\_\mathrm{out}}$
	LR-based	90.25%	92.5%	89.25%	94.25%	92.5%	86.5%	86.25%	89.75%	94.5%	95.0%	94.25%
	FC-based	91.25%	96.75%	92.25%	96.0%	95.5%	92.25%	93.5%	94.0%	96.75%	97.5%	96.75%
	LSTM-based	95.25%	98.75%	93.0%	95.5%	97.5%	96.75%	95.25%	98.5%	98.5%	99.25%	97.25%
